# A review of sourdough starters: ecology, practices, and sensory quality with applications for baking and recommendations for future research

**DOI:** 10.7717/peerj.11389

**Published:** 2021-05-10

**Authors:** Martha D. Calvert, Anne A. Madden, Lauren M. Nichols, Nick M. Haddad, Jacob Lahne, Robert R. Dunn, Erin A. McKenney

**Affiliations:** 1 Department of Food Science and Technology, Virginia Polytechnic Institute and State University (Virginia Tech), Blackburg, VA, United States of America; 2 Department of Applied Ecology, North Carolina State University, Raleigh, NC, United States of America; 3 Kellogg Biological Station and Department of Integrative Biology, Michigan State University, Hickory Corners, MI, United States of America; 4 Center for Evolutionary Hologenomics, University of Copenhagen, Copenhagen, Denmark

**Keywords:** Sourdough, Ecology, Artisanal, Baking, Sensory, Bread, Bacteria, Yeast, Fermentation

## Abstract

The practice of sourdough bread-making is an ancient science that involves the development, maintenance, and use of a diverse and complex starter culture. The sourdough starter culture comes in many different forms and is used in bread-making at both artisanal and commercial scales, in countries all over the world. While there is ample scientific research related to sourdough, there is no standardized approach to using sourdough starters in science or the bread industry; and there are few recommendations on future directions for sourdough research. Our review highlights what is currently known about the microbial ecosystem of sourdough (including microbial succession within the starter culture), methods of maintaining sourdough (analogous to land management) on the path to bread production, and factors that influence the sensory qualities of the final baked product. We present new hypotheses for the successful management of sourdough starters and propose future directions for sourdough research and application to better support and engage the sourdough baking community.

## Introduction

Bread-making is an ancient craft that dates back nearly 14,400 years to Neolithic Asia (Arranz-Otaegui et al., 2018) and ancient civilizations from the 26th century BCE (Seibel, 1994; Wood, 1996; Diowksz & Ambroziak, 2007). All leavened bread was once *naturally* leavened; but with the industrialization of bread production, natural fermentation in bread-baking became less common (Diowksz & Ambroziak, 2007). Recently, however, naturally fermented sourdough bread has regained popularity at the industrial, artisan, and home-baking scales (De Vuyst & Neysens, 2005; De Vuyst et al., 2014; Gobbetti et al., 2016; Roesler, 2019; Brown, 2020)—though all such scales of producing sourdough can differ drastically in their development, maintenance strategies, and quality. Nonetheless, the basic component of sourdough products is a sourdough starter, which is a culture of unique and complex microorganisms. Compared to commercially leavened bread, breads and pastries made with sourdough starters have better shelf life as well as various positive nutritional and sensory qualities (Hutkins, 2019).

Reviews of the science of sourdough starters often, but does not always, relate to starter ecology. Many reviews document general trends in sourdough species ecology, especially over the initial development of the sourdough starter culture (De Vuyst & Neysens, 2005; De Vuyst et al., 2014; Gobbetti et al., 2016; De Vuyst, Van Kerrebroeck & Leroy, 2017; Van Kerrebroeck, Maes & De Vuyst, 2017; Carbonetto et al., 2018; Oshiro, Zendo & Nakayama, 2021). Other reviews have discussed fermentation metabolism, competition among species, and flavor formation in sourdough (Hansen & Schieberle, 2005; Gänzle, Vermeulen & Vogel, 2007; De Vuyst et al., 2009; Gänzle, 2014), but this research is minimally relevant for culinary applications. Researchers have also summarized the effects of flour type, geography, and other variables on sourdough starter ecology and sensory outcomes in bread (Minervini et al., 2014; De Vuyst, Van Kerrebroeck & Leroy, 2017), but recent research suggests that there are other, less well-known baking practices that may also contribute to sourdough starter character and bread quality. Lastly, Pétel, Onno & Prost (2017) discuss the volatile profile of sourdough starters and bread products, but they do not link sourdough flavors to microbial ecology, starter maintenance techniques, or other contributory factors. Professional bakers possess an experiential knowledge of sourdough processes that may complement current sourdough research and motivate new research to support the growing artisan bread-baking industry.

We begin with a basic review of what starters are and what they contain. In doing so, we aim to define baking terms for ecologists and ecological terms for bakers. Specifically, we compare microorganisms to trees, starters to plantations, and process parameters to land management. This novel conceptual approach draws from classic ecology examples to provide a framework for investigating sourdough microbial ecology that is broadly relevant to bakers as well as ecologists. Next, we summarize the influence of sourdough starter maintenance practices (which we also refer to as “land management”) on the microbial ecology of starters, followed by a discussion of how both land management and microbial ecology impact the sensory quality of sourdough starters and breads.

Throughout this review, we aim to identify gaps in the current literature and propose new areas of research that are most applicable to the artisanal baking industry, where sourdough is commonly produced. Here we define the artisanal baking industry as occurring at a smaller scale of local distribution compared to industrial commercial enterprises. Our aim with this review is to identify new avenues for research at the interface of ecology, sensory science, food microbiology, and food chemistry in the context of spontaneous sourdoughs (Type 1), while simultaneously providing scientific and practical applications for bakers. We also hope to highlight the need for a common descriptive language for discussing and evaluating sourdough starters and their bread products as well as analysis of sourdough practices in lay culture and artisan baking communities. This review is intended for professional bakers and a broad range of scientists including, but not limited to, those studying food in society through qualitative research methods, sensory science, food ecology, food microbiology, fermentation, and food chemistry.

## Survey Methodology

We systematically searched literature databases using a broad set of keywords and phrases including: (1) sourdough ecology, (2) sourdough bacteria, (3) sourdough yeast, (4) sourdough practices, (5) bakery sourdough, (6) sourdough sensory, (7) sourdough aroma, sourdough flavor, (8) sourdough rheology, (9) sourdough chemistry, (10) sourdough microbiology, (11) sourdough AND [flour type] (e.g., rye, amaranth, spelt, etc.). We focused on research published during the last 20 years, although we included a selection of older foundational publications based on citation frequency. We also include culturally relevant references to bakers themselves, culinary magazines, and popular media.

## Background

Sourdough starters are initiated by allowing bacteria and yeast to colonize and grow in a mixture of water and flour, where they digest carbohydrates to produce a range of compounds that contribute to bread attributes (Hammes & Gänzle, 1998; Carrizo et al., 2016; Carrizo et al., 2017). Yeasts serve as the primary leavening agent in bread products by producing carbon dioxide as a by-product of their metabolism. Bacteria strongly influence starter acidity (by producing organic acids as by-products of their metabolism), volatile organic compounds, and other characteristics. The specific microbial taxa present in a sourdough starter, and all the dimensions of their biodiversity, can depend on increases and decreases in populations of particular species due to stochastic (random) rather than deterministic factors. They also depend on dispersal processes (Vellend, 2010) associated with which species and strains happen to colonize a starter initially (Gänzle & Ripari, 2016). However, the processes most relevant to bakers are those that are deterministic and associated with niche-related processes. These processes are influenced by conditions intrinsic to the starter itself, including the ingredients (e.g., flour and water) and the recipe. They are also influenced by extrinsic factors, such as baking practices, storage temperature, the amount of starter used during propagation, the number of propagation steps, and fermentation time.

Sourdough microorganisms, in turn, have the potential to influence the ecosystem functions and processes of the starter in ways that can ultimately influence the final bread. Shifts in the ecology of a starter are reflected in the physical characteristics of the starter, raw bread dough, and cooked bread product; which include the technical properties of the dough (i.e., extensibility, elasticity, viscosity) and the organoleptic properties (i.e., bread volume, crumb texture, flavor) of the final bread (Clarke et al., 2004; De Vuyst & Neysens, 2005; Minervini et al., 2014; De Vuyst et al., 2014; Fujimoto et al., 2019). These features of bread comprise what ecologists refer to as ecosystem functions (for those features that have an end state, such as texture) and ecosystem processes (for those features that are measured as rates, such as rise; Minervini et al., 2014).

**Figure 1:**
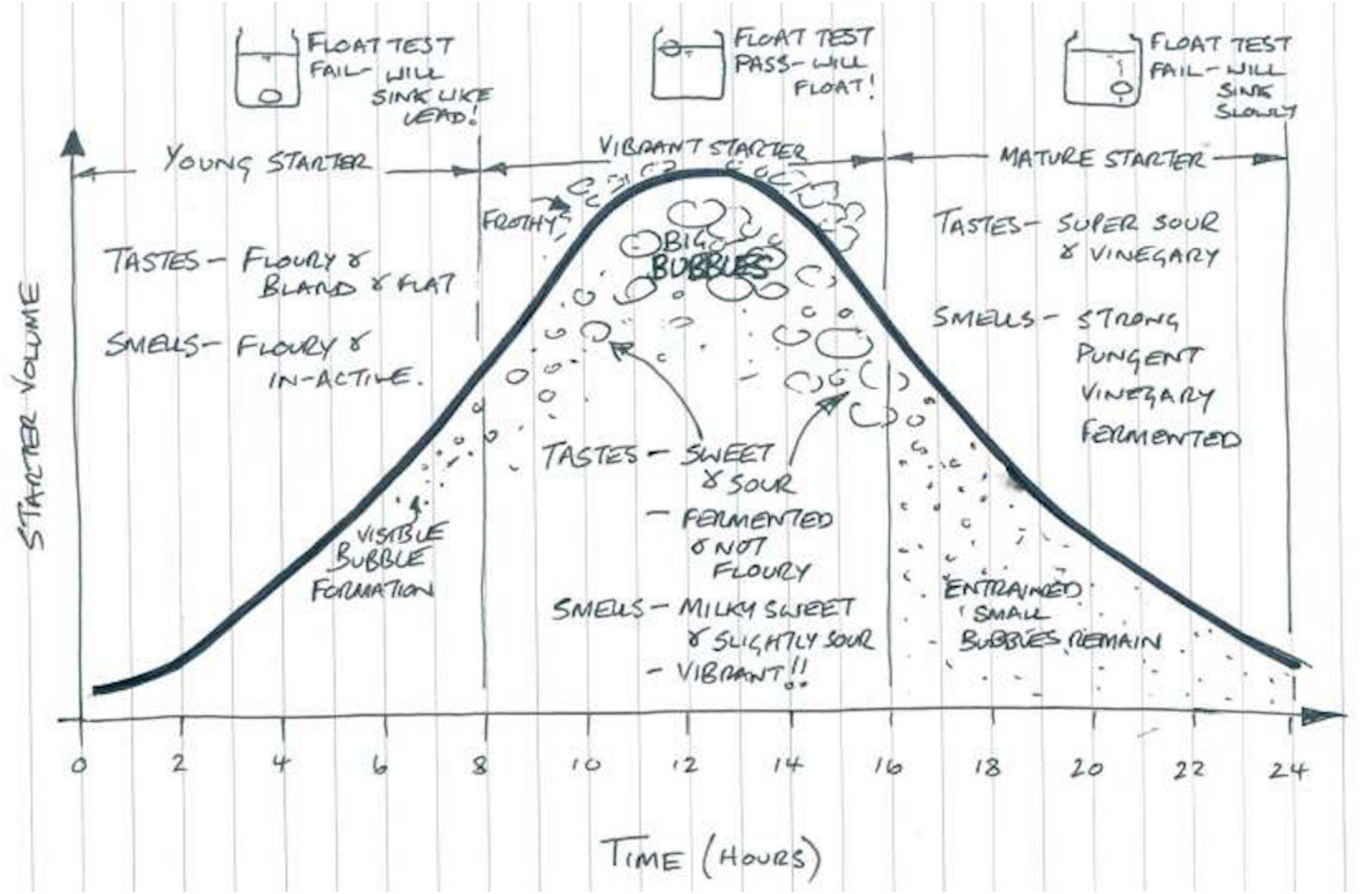
Sourdough starter maturity, as described by bakers (©2015 Adam Veitch, used with permission). A young starter is not yet dominated by well-adapted microorganisms, will lack complex flavors, and will not be a strong leavening agent if used in bread. Vibrant starters have big bubbles and sweet/sour tastes and aromas resulting from fermentation. A mature starter has just surpassed its peak of fermentation and is readily becoming nutritionally deficient for fermenting microorganisms, as characterized by excessive acid-related flavors and smaller air bubbles or a collapsed structure overall.

One can characterize different sourdough starters by their physical environment (as in ecosystems) or by their species composition (as in ecological communities of species or strains). With regard to their physical environment, young sourdough starters are characterized by high carbohydrate availability; but mature starters are more dynamic environments characterized by high CO_2_ production and limited nutrients (De Vuyst & Neysens, 2005; De Vuyst, Van Kerrebroeck & Leroy, 2017). With regard to their species composition, there are four primary types of sourdough starters. Type I sourdough starter communities result from spontaneous succession in the same way that a forest might grow from a fallow field. That is, environmental bacteria and yeasts colonize a flour-water mixture and ferment the digestible carbohydrates in the mixture (along with other nutrients). The community is provisioned with resources over the course of 5–15 days through continuous refreshment with flour and water, also known as backslopping or propagation, during which part of the mixture is discarded (De Vuyst & Neysens, 2005; Gobbetti et al., 2016). Type I sourdough starters are most commonly used in artisanal bakeries and are usually kept at ambient temperature (20–30 °C), though they can be refrigerated when not in use or at regular intervals. These starters usually begin with a near-neutral pH, which steadily declines until maturation. Mature Type I starters are highly acidic due to organic acid produced by lactic acid bacteria (LAB) and acetic acid bacteria (AAB). Macroscopic observations (i.e., amount of bubbles, volume/height, smell, flavor) to define a *mature* Type I sourdough starter are not well-described in scientific literature, and Gobbetti et al. (2016) reports that true Type 1 starters have not been made at the industrial scale. Bakers traditionally determine a starter’s maturity by assessing its sensory characteristics (i.e., visual appearance or flavors), as shown in Fig. 1. In scientific research, starter maturity is generally assessed via the stability of the starter’s pH, rise, or microbiota—in which certain species of bacteria and yeasts consistently appear at certain times (i.e., youth or maturity).

Type II sourdough starters involve inoculating specific bacteria (usually LAB) and/or yeast (i.e., baker’s yeast) into a flour-water mixture. Other species can colonize these mixtures, but they are typically less abundant, and any inoculated microorganisms will ultimately supersede the naturally present microorganisms (from flour or water; De Vuyst, Van Kerrebroeck & Leroy, 2017). Type II sourdough starters are akin to tree plantations, in which a desired tree species is planted but where additional species might also colonize randomly. Compared to Type I starters, Type II starters are usually fermented at higher temperatures for a quicker build, favoring bacterial organic acid production and ultimately resulting in a lower pH system (De Vuyst & Neysens, 2005; De Vuyst, Van Kerrebroeck & Leroy, 2017; Siepmann et al., 2019). While Type II starters are easier to use at large scales of production because of their consistency, they impart specific flavor profiles and can produce a different crumb texture or structure, or loaf volume (Guerzoni et al., 2007; Sterr, Weiss & Schmidt, 2009; Bender et al., 2018; Gaglio et al., 2018). Expanding on the analogy of trees in plantations, specific bacteria are added to Type II starters to achieve unique (i.e., extra sour) organoleptic properties and enhanced shelf life (Gänzle & Zheng, 2019), because ultimately the dough will be supplemented with bakers’ yeast (or any other yeast which is acid-tolerant and an extreme CO_2_ producer) to ensure a full, adequate rise. In contrast, artisan, craft, and home bakers do not experiment with starter cultures of specific microorganisms for their desired outcome; rather, they consistently make and maintain a spontaneous starter culture without knowing anything about the microbial constituency or its functions.

Type III and Type IV sourdough starters are unique extensions of Type I and Type II starters in the culinary industry and in industrial bread production. Type III sourdough starters are dried versions of Type II sourdough starters (De Vuyst & Neysens, 2005; Siepmann et al., 2019). They do not have a direct analogue in terrestrial ecosystems: rather, they may be akin to bags of mixed seed or seed banks in fire or drought prone habitats. Type III starters are unique in that, compared to Type II starters, they can be more easily stored and transported in the dried format.

Type IV sourdough starters are inoculated (Type II) starters that are maintained according to traditional (Type I) methods (De Vuyst et al., 2014; Siepmann et al., 2018), though they have also been referred to as Type 3 (as opposed to Type III) starters (De Vuyst, Van Kerrebroeck & Leroy, 2017). Type IV starters can also refer to the addition of adjuncts, such as fruit or honey, in both Type I or Type II starters (Siepmann et al., 2018; Siepmann et al., 2019). In doing so, Type IV starters challenge the effects of ecological drift and dispersion on the sourdough ecosystem by forcing competition between inoculated microorganisms and those naturally present in the local sourdough environment (Siepmann et al., 2018). Altogether, the microbial ecology, organoleptic qualities and leavening potential of Type III and Type IV starters, especially at the artisan scale, are less understood (Lattanzi, Minervini & Gobbetti, 2014). Our review will focus on traditional (Type I) sourdough starters, which are the most common type used in bakeries and homes.

## Microbial Ecology

Compared to many well-studied environmental and bodily systems (i.e., soil or guts; Fierer, 2017), sourdough starters are relatively low in microbial diversity, especially phylogenetic diversity. Sourdough therefore provides a relatively simple system in which to examine and test theories relevant to microbial ecology (Landis et al., 2021). In this section, we examine the taxa that inhabit and characterize different starters, and the rules/processes that govern that membership. Lactic acid bacteria belong to the family *Lactobacillaceae*, which was recently revised such that the names of many LAB have changed (Zheng et al., 2020). Our review reflects this new taxonomy.

### Bacteria

To date, it appears as though sourdough starters initially harbor a modest diversity of bacterial species (hundreds, not thousands) and strains that colonize the starter from diverse sources including human bodies, non-human vertebrates, air, insects, the flour itself and more (Gänzle & Zheng, 2019; Reese et al., 2020). Bacteria are present initially when starting a starter (in the flour, water, or container), and they are added continually over the course of starter maintenance. As the starter matures, the diversity of bacterial genera in the sourdough community decreases (Minervini et al., 2014; Gobbetti et al., 2016). However, the diversity of strains and species that persist, especially LAB, can increase over time (Spicher & Nierle, 1988; Gänzle, Vermeulen & Vogel, 2007; Harth, Van Kerrebroeck & De Vuyst, 2016; Gobbetti et al., 2016). Anecdotally, it appears that beta diversity patterns (i.e., differences among starters) track these changes in alpha diversity (i.e., complexity within starters). Specifically, beta diversity of species and genera is high in young starters, whereas beta diversity of strains within persistent genera is high in more mature starters. The changes in diversity and composition that occur as starters mature are, at least in part, due to the production of organic acids by LAB, which disfavor genera that are not acid-tolerant, but allow the persistence of diverse strains and species of genera that are acid-tolerant (Weckx et al., 2010b; Minervini et al., 2014). LAB can also inhibit the growth of other microorganisms through the production of compounds such as bacteriocins and antifungal peptides (De Vuyst & Leroy, 2007). When individual starters reach maturity, they often contain a mixture of different LAB species and strains of those species. There are more than 60 common species of LAB discussed throughout sourdough literature, including *Fructilactobacillus sanfranciscensis*, *Levilactobacillus brevis*, *Limosilactobacillus fermentum*, *Lactiplantibacillus plantarum*, *Leuconostoc mesenteroides*, *Weissella cibaria*, and *Weissella confusa*; though not all of these species necessarily appear in any given starter (De Vuyst et al., 2014; De Vuyst et al., 2016; Harth, Van Kerrebroeck & De Vuyst, 2016; Gobbetti et al., 2016; Landis et al., 2021). In fact, most mature starter communities tend to be dominated by 1-3 bacterial species (Landis et al., 2021).

The natural history of the most common LAB in sourdough starters, outside of the starter environment is poorly explored. In general, LAB can be found in diverse ecosystems, often in association with animals or plants (George et al., 2018; Yu, Leveau & Marco, 2020). Some LAB species, such as *L. plantarum,* are known to be associated with multiple environments. These generalists are often the first colonizers of spontaneous sourdough starters (Gänzle & Zheng, 2019), perhaps because they are more likely to be present in a given environment. By contrast, some host-adapted LAB species have reduced genomes and even those that do not often have relatively narrower niches than generalists (Zheng et al., 2015; Gänzle & Zheng, 2019). Uniquely, *F. sanfranciscensis* is particularly ubiquitous and dominant across starters (Landis et al., 2021), perhaps in part because it is often associated with insects (including flies and grain beetles) and plants, and therefore may be present in the original flours (Gänzle & Zheng, 2019; Yu, Leveau & Marco, 2020). The potential relationship between the niches of LAB in the wild and their ecology in starters is fascinating but, as of yet, poorly explored. For example, vertebrate associated taxa tend to be thought of as more heat tolerant and plant associates as more cold tolerant.

**Figure 2:**
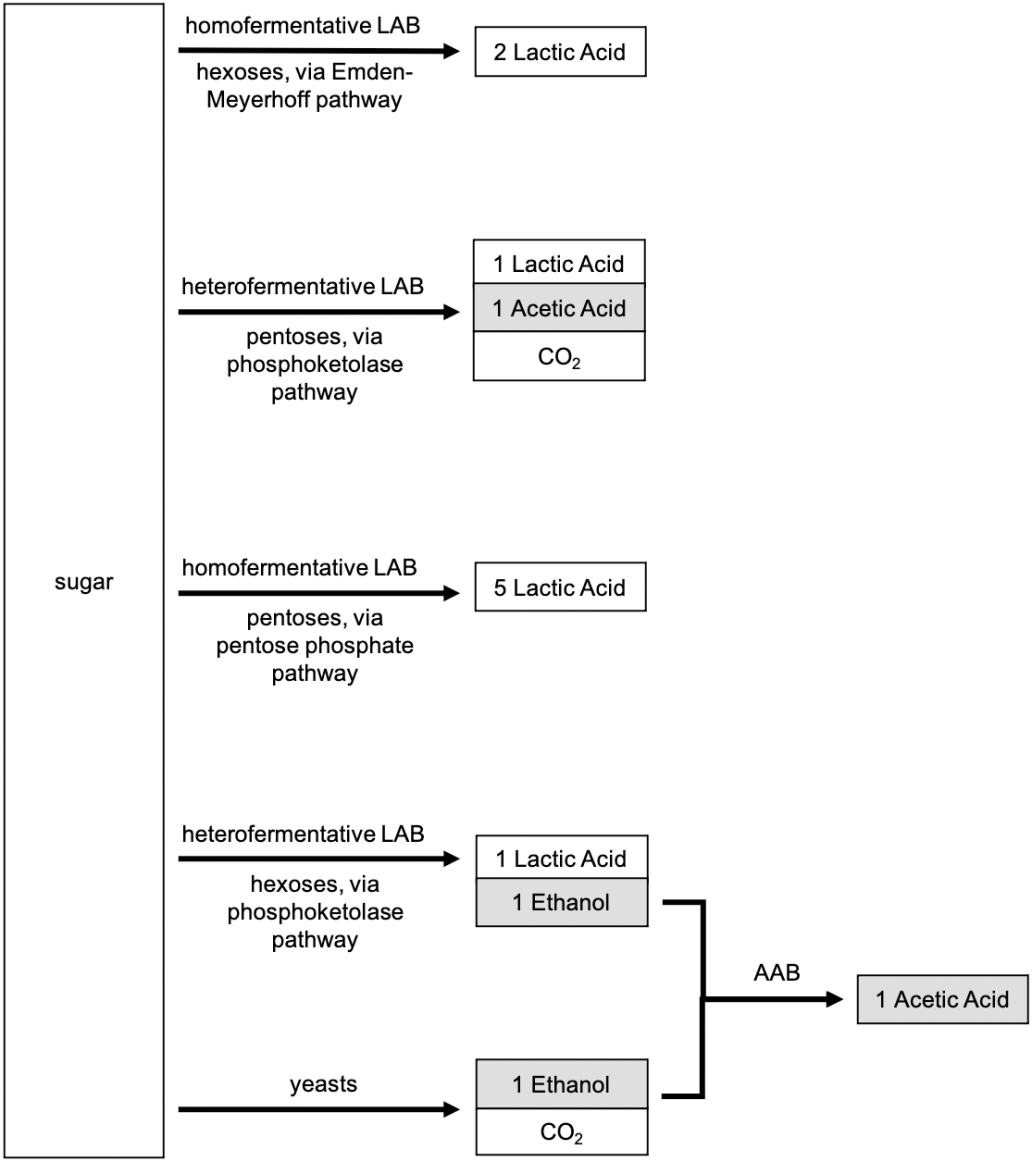
Metabolic products associated with lactic acid bacteria (LAB) and yeasts. Heterofermentative pathways adapted from Gänzle (2015).

LAB can also be categorized as a function of their energy metabolism: whether they are heterofermentative (*hetero-*, producing more than one byproduct during fermentation) or homofermentative (*homo-*, producing one byproduct during fermentation). A brief metabolic flow chart distinguishing the different microbial metabolisms is shown in Fig. 2. The relative abundance, activity, and diversity of heterofermentative versus homofermentative LAB can greatly influence the functions and processes of sourdough starters because heterofermentation produces lactic acid, acetic acid, ethanol, and CO_2_, while homofermentation produces only lactic acid (Gänzle, 2015; Zheng et al., 2015). Thus, heterofermentative metabolism contributes more to dough rise compared to homofermentative species, supplementing leavening due to yeasts through the production of additional carbon dioxide. Mature sourdough starters are typically dominated by heterofermentative LAB (De Vuyst & Neysens, 2005). Yet, much remains to be learned: metabolic processes of heterofermentative LAB can vary between strains of bacterial species within a given starter, as described for *L. plantarum* studied by Häggman & Salovaara (2008b). Identifying and selecting microorganisms with specific metabolisms is relevant for developing Type II starters with specific characteristics; but, bakers that utilize Type I starters do not have the ability to identify bacterial metabolisms present in their starter nor to select for sensory characteristics in their sourdough products based on metabolism (i.e., bakers cannot deliberately increase the abundance of heterofermentative LAB, or know when heterofermentative LAB are dominant, in a spontaneous starter). We propose that future works aim to identify which modes of metabolism dominate under specific conditions, and how different metabolisms lead to observable sensory qualities in sourdough products.

Acetic acid bacteria have been briefly described in sourdough starters, though they are generally less prevalent and less abundant in Type I starters compared to LAB, and the factors that influence their presence in sourdough starters are not well understood (Di Cagno et al., 2014). Research by Comasio et al. (2020) found that starters kept in Lambic breweries (where open fermentation vessels were kept nearby) harbored more diverse AAB, supporting our discussion (section 3.6) of how the local environment can influence a starter’s ecology. They also observed that AAB were negatively associated with *F. sanfranciscensis*, but positively associated with some yeasts. Yet, the specific interactions between LAB, AAB, and yeast in starters are poorly characterized. In some contexts, AAB may contribute unpleasant aromas to sourdough starters (Landis et al., 2021). However, their role may depend on the grain being fermented. For example, it has been argued that AAB may play key roles in the development of gluten-free sourdough bread products (Ua-Arak, Jakob & Vogel, 2016; Ua-Arak, Jakob & Vogel, 2017).

#### Organic acids produced by bacteria

Organic acids are one of major byproducts of LAB and AAB fermentation that affect the gluten network structure, dough elasticity, and various sensory properties that distinguish sourdough products from other commercially leavened baked goods (Clarke et al., 2004; De Vuyst et al., 2009; Siepmann et al., 2019; Moroni et al., 2012; Van Kerrebroeck et al., 2018). The quantity and type of organic acids in a starter result from the starter’s bacterial ecology, but the dominant type of organic acid in a starter is also a function of starter maintenance practices including fermentation time, temperature, and dough yield (Decock & Cappelle, 2005; Minervini et al., 2012a; Di Cagno et al., 2014). Whereas lactic acid is produced by both homo- and heterofermentative LAB, acetic acid is also produced by AAB. In traditional sourdough starters (Type I), lactic acid levels usually supersede acetic acid levels, and AAB are less dominant than LAB in spontaneous sourdough cultures (Landis et al., 2021). However, bacterial organic acid production and tolerance also varies among species and strains of LAB, AAB, and even yeasts (Suihko & Mäkinen, 1984; Gänzle, Ehmann & Hammes, 1998; Brandt, Hammes & Gänzle, 2004; Häggman & Salovaara, 2008a; Sterr, Weiss & Schmidt, 2009; Minervini et al., 2012a; Hutkins, 2019; Carbonetto et al., 2020).

The relationship between bacterial organic acid production and yeasts’ CO_2_ production is complex but relevant for bakers. Researchers have proposed that fermenting sourdough starters at low temperatures for a long time will produce more acetic acid and amplify the iconic tangy flavors of sourdough products (Spicher & Brümmer, 1995; Hansen & Hansen, 1996; Hammes & Gänzle, 1998; Gobbetti et al., 2000; Osimani et al., 2009; De Vuyst et al., 2016). Increasing organic acid production requires acid-tolerant species; and decades of sourdough research have focused on increasing organic acid production in inoculated sourdoughs (Type II) which are ultimately supplemented with baker’s yeast (*Saccharomyces cerevisiae*) or acid-tolerant yeasts for leavening. Many researchers associate acetic acid with increased yeast activity because yeast liberate fructose, which heterofermentative LAB then use to make acetic acid (Decock & Cappelle, 2005; Häggman & Salovaara, 2008a; Corsetti, 2013; De Vuyst et al., 2014; De Vuyst et al., 2016; De Vuyst, Van Kerrebroeck & Leroy, 2017; Siepmann et al., 2019). Yet, acetic acid is also thought to inhibit yeast more than does lactic acid in both inoculated and bakery sourdough starters (Moon, 1983; Hellemann et al., 1988; Narendranath, Thomas & Ingledew, 2001; Minervini et al., 2012a; De Vuyst et al., 2014; Lattanzi, Minervini & Gobbetti, 2014).

Studies of spontaneous bakery starters note that refrigeration temperatures are in fact associated with decreased leavening activity (Di Cagno et al., 2014), and that increased acetic acid levels correlate to lower yeast cell densities (Minervini et al., 2012a). By contrast, Lattanzi, Minervini & Gobbetti (2014) observed that starters with low acid (particularly low acetic acid) were dominated by yeasts. Furthermore, low-pH or cold-fermented sourdough starters may only be viable leavening agents when supplemented with bakers’ yeast or when dominated by strictly acid-tolerant microorganisms, such as *Kazachstania humilis* (Häggman & Salovaara, 2008a; De Vuyst et al., 2016).

When a Type I starter is used as the sole leavening agent in sourdough bread, its acidity must therefore be controlled to best support yeast activity. In practice, a mature Type I sourdough starter that is past its peak becomes overly tart, acidic, and pungent, and produces fewer air bubbles (i.e., decreased CO_2_ production; Fig. 1). A starter’s organic acid-related flavors can enable a baker to diagnose how vibrant or “ready” a starter is for use in traditional baking. Yet, bakers may struggle to decipher if the organic acid in their sourdough products is heavily lactic acid or acetic acid (Hellemann et al., 1988), and few studies discuss how the baking process can and should be adjusted, to modify or accommodate the organic acid profile or acidity of a starter. Because artisanal (Type I) sourdough bread baking requires balancing flavor and structure at the mercy of organic acid, we propose that sensory research further investigate the connections between sourdough acidity, ecology, and maintenance practices. We discuss these further throughout section 3 and 4.

### Yeast

A typical starter contains one hundred times as many bacterial cells as yeast cells (a 100:1 ratio), though the ratio of LAB to yeast can range from 1:1 to 1000:1 (De Vuyst & Neysens, 2005; Minervini et al., 2012a). Yeasts are single-celled fungi able to clonally reproduce but unable to produce true hyphae, making them well-suited for bread-making. As we have mentioned, their critical role in the sourdough environment is producing CO_2_ for leavening. But yeasts also work synergistically and antagonistically alongside LAB, producing various classes of alcohols, esters, and organic acids that impact both bacteria activity and sourdough flavor (Corsetti, 2013; De Vuyst et al., 2016; Carbonetto et al., 2018). Indeed, yeasts compete for survival in the sourdough ecosystem by producing antimicrobial substances (which act as allelochemicals; Guerzoni et al., 2007; Hatoum, Labrie & Fliss, 2012). Generally, yeasts are sensitive to environmental conditions (Van Kerrebroeck, Maes & De Vuyst, 2017; Michel et al., 2019), though their resilience over the course of a sourdough starter’s age is uncertain. Throughout our global history of baking, yeasts have evolved to favor rapid fermentation and maximum CO_2_ production (Bigey et al., 2020).

Sourdough yeasts include species of diverse genera within the order *Saccharomycetales*. Our review of the literature identified ∼40 different species of yeasts that have been described in the sourdough environment, with some species being first discovered and named from sourdough starters (Jacques et al., 2016; De Vuyst et al., 2016; Carbonetto et al., 2018); more recently, Landis et al. (2021) identified ∼70 variants across 500 starters collected from 17 countries around the world. However, of this *potential* diversity (i.e., gamma diversity), the average starter typically contains relatively few species—in many cases only one or two, sometimes no yeasts at all, and sometimes more (De Vuyst et al., 2014; De Vuyst et al., 2016; Viiard et al., 2016; Michel et al., 2019; Landis et al., 2021). As a result, the alpha diversity of yeasts in starters (i.e., the average diversity per starter) is low, but the gamma diversity (i.e., the regional diversity pool across all starters) and beta diversity (i.e., the difference in yeasts from starter to starter) are relatively high.

Common sourdough yeasts include but are not limited to *Saccharomyces cerevisiae*, *Kazachstania humilis*, *Candida krusei*, *Kazachstania exigua*, *Torulaspora delbrueckii*, *Wickerhamomyces anomalus,* and *Pichia kudriavzevii* (Ercolini et al., 2013; Liu et al., 2018). Outside of the sourdough environment, many of these yeasts are similar in that they rely on insects for dispersal (Stefanini, 2018; Madden et al., 2018; Bigey et al., 2020). *S. cerevisiae* is often considered to be the “canonical” sourdough yeast because it is ubiquitous across most starters and often associated with the first leavened breads (Valmorri et al., 2010; Landis et al., 2021). However, because *S. cerevisiae* reproduces rapidly when compared with other yeast species (Landis et al., 2021), its ubiquity may be relatively recent and a reflection of rapid dispersal and invasion facilitated by human movement among bakeries and continents (Minervini et al., 2012a; Michel et al., 2019; Bigey et al., 2020).

Much like bacteria, the origin of yeast in the sourdough ecosystem is thought to be related to various factors, such as the flour or the immediate environment in which a starter is kept (i.e., home kitchen versus commercial bakery); these factors are further discussed in section 3.1–3.7. Indeed, yeasts may colonize starters from vessels, implements, and even from the bodies of bakers (Reese et al., 2020), as is the case with the fast-growing *S. cerevisiae* (De Vuyst & Neysens, 2005; De Vuyst et al., 2009; De Vuyst et al., 2016; Minervini et al., 2014; Gänzle & Ripari, 2016). Yet, regardless of their origin, the time frame over which yeasts appear in starters varies: yeasts can dominate over bacteria at the beginning of propagation, or they may not appear until days 5–7 of propagation (Moroni, Arendt & Bello, 2011; Ercolini et al., 2013). The factors that influence yeast colonization in the initial build of a starter, the rate of yeasts’ assimilation to the starter culture concurrent with environmental changes, and the successful activity of individual species compared to others are all growing areas of research.

### Diversity

Throughout the history of ecology, scientists have investigated the effects of species diversity on ecosystem function (Elton, 1958). Advances in this arena have accelerated in the last two decades, in association with experimental studies in which the number of species is controlled (Tilman et al., 1997; Tilman, Isbell & Cowles, 2014). At a gross level, ecologists have investigated the effects of functional diversity—for example, among plant groupings of grasses, forbs, and legumes (Tilman et al., 1997). Are similar relationships between microbial diversity and ecosystem functions observed in sourdough? Sourdough communities might be considered to contain two functional groups: yeast and bacteria. These functional groups play different roles (e.g., production of ethanol and organic acids, respectively) that significantly impact ecosystem function (i.e., starter performance and bread attributes). For example, Landis et al. (2021) detected consistent pairwise interactions between dominant bacterial and yeast species that may contribute to overall community structure.

Ecologists ask: after accounting for functional diversity, does the diversity of species (i.e., richness) affect ecosystem function? In plant communities, the answer is yes (Tilman, Isbell & Cowles, 2014). In microbial communities, the strongest functionally relevant aspect of diversity may be strain diversity. For example, we describe in section 2.1 how fermentation metabolism varies across different strains of *L. plantarum*. There is also evidence of vast intra-species diversity in yeasts such as *Wickerhamomyces anomalus* (Daniel et al., 2011), *Kazachstania humilis* (Carbonetto et al., 2020), and *Saccharomyces cerevisiae* (Valmorri et al., 2010; Bigey et al., 2020). Valmorri et al. (2010) found *S. cerevisiae* in 16 of 18 sourdough starters from Italy and categorized the various strains into seven different phenotypic groups. However, while we know that intra-strain diversity exists within microbial species, we cannot yet compare the functional differences within species because of confounding variables including differences in where and how starters are made and maintained. These cases are analogous to the strong effects found in plant genetic diversity that, surprisingly, can be equivalent to plant species diversity (Crutsinger et al., 2006).

By what mechanisms can diversity of strains, species, or functional groups affect the function of sourdough ecosystems? Different strains, species, or functional groups fill different niches that allow the organisms within each niche to exploit available resources in different ways, serving to increase functional response (Loreau & Hector, 2001). For example, different sourdough microorganisms preferentially metabolize different carbohydrates to different products (Fig. 2; Hammes & Gänzle, 1998; De Vuyst & Neysens, 2005; Carrizo et al., 2016; Carrizo et al., 2017). This is one example of how species diversity and interactions between species, both mutualistic and antagonistic, can be key to maintaining stable community dynamics (Mougi & Kondoh, 2012; Gracia-Lázaro et al., 2018; Landis et al., 2021). Different starter communities produced distinct aromatic compounds and profiles, which would presumably affect bread taste and aroma (Landis et al., 2021). The effects of diversity thus extend to ecosystem response and, in turn, sourdough attributes perceived by human consumers.

A central theme of our paper is the role of microbial diversity on sourdough ecosystem function and characteristics, as mediated by the physical environment. Biological and physical factors are modified in hundreds of ways, all over the world. The sourdough system is ripe for controlled experiments to test for mechanisms of species diversity’s effects once all of the variables of sourdough production are accounted for.

### Succession

Many studies have considered succession (i.e., the process by which community membership changes over time) in grasslands (Archer et al., 1988), old fields (Keever, 1950; Myster & Pickett, 1994) and other wild ecosystems. In those studies, the first species to colonize will help facilitate the next wave of colonizing species, and the final species in a mature ecosystem inhibit the colonization of other species (once a climax community is reached). In wild nature, such clean successional dynamics are rare, but they seem to be the norm in sourdough starters.

Succession in sourdough cultures is well-described in the literature to include three phases: (1) an influx of diverse microbial species related to that which is naturally present in the flour, (2) an increasing presence of sourdough-specific genera (e.g., *Fructilactobacillus, Lactobacillus*, *Companilactobacillus, Ligilactobacillus, Levilactobacillus, Lentilactobacillus, Lacticaseibacillus, Limosilactobacillus, Pediococcus* and *Weissella*), and (3) a dominance of well-adapted sourdough species, primarily heterofermentative species (De Vuyst & Neysens, 2005; Weckx et al., 2010a; Ercolini et al., 2013; Minervini et al., 2014; Harth, Van Kerrebroeck & De Vuyst, 2016; Gobbetti et al., 2016). As the bacterial ecology of a starter changes on the path to maturity, the acidity of the sourdough culture increases, selecting for acid-tolerant bacteria and yeast (Ravyts & De Vuyst, 2011; De Vuyst et al., 2014). As in wild grasslands, a mature starter’s final ecology hinders colonization by new species (introduced during refreshments) in ways that are not yet clear, but closely connected to organic acid production and other competitive behaviors. While some researchers have explored microbial succession in starters over time (Minervini et al., 2018), we do not conclusively know how the long-term age of a starter affects its microbial community and functional characteristics. Exploring the relationship between a starter’s age and its microbial ecology, resiliency to land management changes, and sensory quality may be an interesting direction for future research.

There is evidence that the three-phase succession of species occurs according to a different timeline in starters containing non-wheat flour (Sterr, Weiss & Schmidt, 2009; Weckx et al., 2010b; Rodríguez et al., 2016; Bender et al., 2018). More work is needed to compare the succession of both bacterial and fungal membership in sourdough starters across time and all commonly used flour types. Further, understanding which maintenance factors and environmental stressors impact the ecological evolution of starters may reveal ways to create more stable starters in artisanal bakery settings.

## Sourdough Starter Maintenance as “Land Management”

Sourdough propagation practices include all of the technical processes that are employed during the initial development of a sourdough starter and in the subsequent maintenance of the starter. In forests and grasslands, these maintenance practices comprise what tends to be called “land management”. De Vuyst, Van Kerrebroeck & Leroy (2017) also discuss land management practices, referring to them as ‘process parameters.’ They note that studying starter land management is challenging in artisanal or home baking because practices can range drastically and change often. In this way, the practice and knowledge of bakers with regard to management of sourdough starters is a form of traditional ecological knowledge. The land management of starters can potentially alter the initial composition of microorganisms, the dynamics of succession, and/or the final proportions of the microorganisms present. Inasmuch, land management can also affect the metabolism and performance of the starter. Sourdough research to date suggests that the most influential management factors are (1) flour type, (2) hydration, (3) temperature, (4) time, (5) re-feeding practices, (6) environment, and (7) other factors, which we now consider in turn.

### Flour

The type of flour that is used during starter maintenance can impact the culture’s ecology, technical effectiveness as a leavening agent, and unique sensory qualities. Flour helps to introduce microorganisms into the sourdough medium (Reese et al., 2020). Flour also supplies different nutrients (i.e., carbohydrates and amino acids) and non-nutrients (i.e., phenolic acids, amylase, ash), the presence and concentrations of which can influence bacteria and yeast species survival (Vogelmann & Hertel, 2011; Minervini et al., 2014; Minervini et al., 2018; Gänzle, 2014). For example, different bacteria and yeast preferentially digest different saccharides, which can influence species co-occurrence through either mutualism or antagonism (Gracia-Lázaro et al., 2018). Specifically, *F. sanfranciscensis* can use maltose as a substrate (and hence is said to be “maltose positive”) and is often found with *Kazachstania humilis* which cannot metabolize maltose (De Vuyst et al., 2014). However, carbohydrate preference is not the only factor influencing stable species interactions in the sourdough ecosystem (Gracia-Lázaro et al., 2018; Carbonetto et al., 2020).

Non-nutrients also contribute to the carbohydrate availability and hence microbial ecology within the sourdough medium. For example, the amylase enzymes present in grains and flour help break complex carbohydrates down into fermentable carbohydrates, favoring rapid bacterial fermentation of the sourdough culture and forcing the colonization of acid-tolerant species over a shorter fermentation period (Gänzle, 2014; Yildirim-Mavis et al., 2019). Flour types differ in their concentrations of amylase. We hypothesize that high-amylase systems (e.g., rye flour) yield sourdough starters that acidify quickly; this may lead to reduced yeast activity, stronger sour flavors, or even increased shelf life, but it could also be combated with more often re-feedings.

Phenolic compounds and ash are naturally present, to varying degrees, in flour and they have also been shown to affect the acidification rate of starters, thus impacting microbial succession (Gobbetti et al., 1995; Gänzle, 2014; Pétel, Onno & Prost, 2017; Koistinen et al., 2018). Similarly, the presence or absence of the bran portion of grain may also influence microbial ecology in ways that are not yet clear (Corsetti et al., 2007).

The different organisms that typify starters fed with different flours are the focus of a growing body of research. Different varieties of wheat have even been shown to support different sourdough ecologies, and non-traditional wheat varieties (i.e., einkorn and emmer) are also growing in popularity in the culinary industry (Coda et al., 2010; Minervini et al., 2012a; De Vuyst et al., 2014). However, studies which explore the sourdough ecology and flour connection have used wildly different approaches (Succi et al., 2003; Van der Meulen et al., 2007; Vrancken et al., 2010; Paramithiotis, Tsiasiotou & Drosinos, 2010; Moroni, Arendt & Bello, 2011; Ercolini et al., 2013; Bessmeltseva et al., 2014; Zheng et al., 2015; Lhomme et al., 2015; Harth, Van Kerrebroeck & De Vuyst, 2016; Carrizo et al., 2016; Van Kerrebroeck et al., 2018). Understanding the association between flour type, microbial ecology, and sensory quality in sourdough products is challenging because many other starter management techniques should be controlled; thus, we emphasize that the impact of flour type on a starter’s microbial ecology and efficacy as a leavening agent is still unclear (De Vuyst, Van Kerrebroeck & Leroy, 2017). Future research should aim to better understand and define sourdough land management in culinary practice, and then use those standard methods to assess the microbial ecology, flavor, and leavening activity of starters containing unique flours. We emphasize this further in the following sections.

### Hydration

In terrestrial ecosystems, the availability of water is a key factor governing species distributions, ecological dynamics and other phenomena. Similarly, the water available in a sourdough starter is important to the ecology of the starter. The water available in the starter is referred to as hydration (i.e., the percent of water to flour) or dough yield (i.e., the ratio of flour to water when using 100 grams of flour). Dough yield is most often referenced in scientific literature, but hydration is most often discussed among the traditional baking community. Low hydration, low dough yield, and firm or stiff starters all describe systems with lower amounts of water. Hydration is relevant to microbial ecology and their metabolic function because water diffuses nutrients and proteolytic enzymes (Hager et al., 2012; De Vuyst et al., 2014; De Vuyst, Van Kerrebroeck & Leroy, 2017; Akinola & Osundahunsi, 2017). A starter’s hydration also influences the activity and composition of microorganisms in starters. Generally, it is thought that liquid starters favor more LAB activity and stiff (dry) starters favor more yeast, but this model is not conclusive across all starters; (Minervini et al., 2014; Di Cagno et al., 2014). For example, LAB such as *L. plantarum* and *F. sanfranciscensis* have been observed to dominate in stiff starters (De Vuyst & Neysens, 2005; Minervini et al., 2012b; Di Cagno et al., 2014). Generally, scientists discuss the acid-ecology relationship more than the hydration-ecology relationship (De Vuyst, Van Kerrebroeck & Leroy, 2017); yet bakers more easily and objectively measure hydration than they subjectively and sensorially measure acidity. We suspect that the role of hydration is better understood by professional bakers because they produce starters at larger scales and on a more consistent basis, and bakers may have a broader understanding of how their starter’s hydration impacts the quality of their bread. Baking practices and anecdotes may inform future research and scientific knowledge of the mechanisms by which hydration impacts the bacteria to yeast ratio, microorganism-related sensory qualities, and the leavening potential of sourdough breads.

### Temperature

The fermentation temperature and storage temperature of a starter are said to account for 44% of a sourdough bread’s physical, chemical, and volatile characteristics via the effects of temperature on microbial activity (Siepmann et al., 2019). This being said, dough is like nature more generally, where temperature has a disproportionate effect on the number and identity of species in ecosystems (Currie, 1991). The “temperature of a starter” and the “fermentation temperature”, actually refer to several temperatures: (a) the temperature at which the starter is fermented, (b) the temperature at which it is re-fed, and (c) the temperature at which it is used in bread recipes. These different temperatures can vary independently. The temperature at which a starter is fermented (a) can then also include both room temperature and refrigerated temperatures over the course of a daily feeding cycle. In addition, bakers may store a starter in the refrigerator between feedings but allow for a “resting period” at room temperature before re-feeding or using in a bread recipe. Bakers may even modify the temperatures of ingredients to control the rate of fermentation. For example, in summer months bakers may use extremely cold ingredients such as ice water and refrigerated flour to slow the pace of a “hot” fermentation. The temperature that a starter is fermented at is often specified in scientific literature, but storage temperatures across the entire fermentation period are not thoroughly documented across bakery research (Di Cagno et al., 2014; Lhomme et al., 2015; De Vuyst, Van Kerrebroeck & Leroy, 2017).

In soil microbial ecosystems as well as plant and animal communities, temperature strongly influences which species survive (Jeffree & Jeffree, 1994), ecological functions, and ecosystem processes (Rasmussen et al., 2011). The same appears to be true for sourdough starters, though much remains to be studied. Temperature influences microbial dominance (bacteria vs. yeast), growth (cell density), and microbial activity (production of organic acid, volatiles, and CO_2_) based on seasonal fluctuations and characteristics of the baking environment (De Vuyst et al., 2014; Viiard et al., 2016; De Vuyst, Van Kerrebroeck & Leroy, 2017). It is frequently suggested that warmer fermentations (>30 °C) favor bacterial activity and organic acid production (Hansen & Schieberle, 2005; Sterr, Weiss & Schmidt, 2009; Moroni, Arendt & Bello, 2011; Banu, Vasilean & Aprodu, 2011; Minervini et al., 2014; De Vuyst et al., 2014; De Vuyst et al., 2016; Bessmeltseva et al., 2014; Viiard et al., 2016; De Vuyst, Van Kerrebroeck & Leroy, 2017; Siepmann et al., 2019), but this purported “generality” needs to be better studied. There is also evidence to suggest that warmer sourdough fermentations (e.g., 20–37 °C) will favor *Lactobacillaceae* compared to cooler fermentations (e.g., 4–15 °C), which can favor *Fructilactobacillus, Leuconostoc,* and *Weissella* species (Minervini et al., 2012b; Harth, Van Kerrebroeck & De Vuyst, 2016).

### Time

Two elements of time are potentially important to sourdough starters. The first element is the age of a starter measured from the time it was developed, akin to “primary” succession in ecology. Starters can be tens or even hundreds of years old; and the day-to-day feeding practices and fermentation times for a single starter can vary, resulting in an ever-evolving microbial system. We suspect that the overall age of a starter influences which species are present in the starter, the day-to-day potential for fluctuations in the ecology of the system, and the genes on which microorganisms rely. For example, Minervini et al. (2018) reported intra-species changes and slightly increasing yeast counts over a one-year period of sampling Italian bakery starters, suggesting that older starters may be better leavening agents. Undoubtedly, this is just one of many kinds of changes that occurs with starters over time.

**Figure 3:**
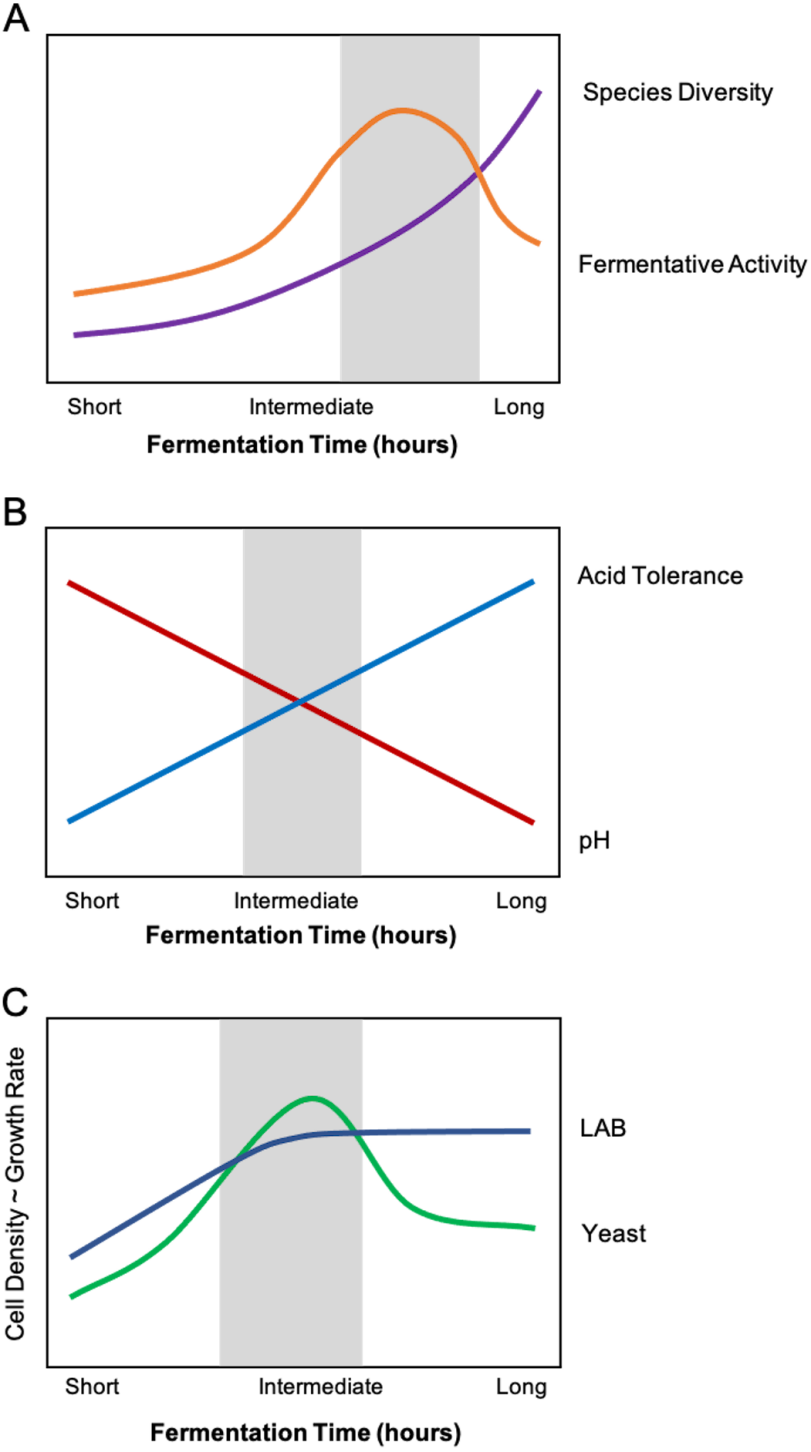
Composite relationships between environmental stressors and microbial growth/function with optimal windows highlighted in grey. (A) An optimal feeding frequency promotes high diversity and fermentative activity. For starters with fermentation times that are too short (i.e., feeding too frequently), the sourdough culture will exhibit suboptimal fermentation activity and slow-growing organisms (i.e., yeasts) will be inhibited. In other words, baking with a starter that has not reached its full fermentative potential may produce bread that is not well-leavened or has an inconsistent crumb texture or structure, among other undesirable sensory qualities. (B) Intermediate fermentation times strike a balance between pH and selection for acid-tolerant microorganisms. Refeeding the starter removes some organic acid from the sourdough system, temporarily relieving acid stress. With longer fermentation times, only acid-tolerant species of lactic acid bacteria (LAB) and yeast will prevail. (C) The ideal fermentation time allows both LAB and yeasts to reach optimal growth rate and cell density. The initial sourdough medium contains slightly more LAB cells compared to yeasts cells. Intermediate fermentation times maximize the growth rate and cell density of both LAB and yeasts. Beyond that window, however, organic acid accumulation inhibits yeast growth and activity.

The second element of time relates to time since feeding, which is often expressed as a frequency of re-feeding (i.e., 12 hours, 24 hours, biweekly). This is akin to “secondary” succession, whereby each feeding/refreshment introduces fresh species from various sources, and all microorganisms need time to adjust to the sourdough culture before reaching their maximum fermentative activity (De Vuyst et al., 2009; Lattanzi, Minervini & Gobbetti, 2014). Disturbance theory suggests that the diversity of microorganisms in starters might vary predictably as a function of feeding/disturbance frequency (Mackey & Currie, 2001; Fox, 2013). We hypothesize that there is a range of feeding frequency which optimizes the fermentative capacity of the sourdough system while maximizing the ecological diversity of sourdough (Fig. 3A). We predict that species diversity is positively correlated with feeding frequency because disturbance favors the growth of opportunistic and generalist species, which can adapt to fill a greater breadth of potential niche space than the specialists that form a climax community over time. Furthermore, if short fermentation time (more frequent feedings) is a consistent practice, then the sourdough culture will eventually select for more fast-fermenting species (Minervini et al., 2014; De Vuyst et al., 2014; Fig. 3A). We might also predict that the relationship between feeding frequency, species diversity, and fermentative activity is closely related to fermentation temperature.

In sourdough research, slow-growing yeasts are often thought to be more likely to succeed with longer fermentation times (lower feeding frequencies), and longer fermentations are likely to harbor more acid-tolerant species (i.e., *L. plantarum*, *L. fermentum*, and *L. reuteri*; De Vuyst et al., 2014; Fig. 3B). However, longer fermentations also give fast-growing bacteria more time to produce organic acids, which can hinder the fermentative activity of species that are not as acid-tolerant (Moon, 1983; Narendranath, Thomas & Ingledew, 2001). We predict that there is an ideal range of fermentation time (which is evident in baking practices) wherein the sourdough ecosystem is balanced in acidity to support both LAB and yeast growth, optimizing leavening and sensory qualities (Fig. 3C). We propose that the presence of acid-tolerant microorganisms will extend to sourdough starters that are refreshed infrequently (i.e., once a month) or even neglected (as in the case of home-baking). Likewise, the “ideal” fermentation time in relation to the quantity of microbial volatile byproducts (i.e., organic acids, ethanol, etc.) is subjective, as some bread bakers may prefer the style of pungent, tart sourdough breads resulting from extended fermentation.

### Re-feeding practices

Re-feeding a starter is the process of discarding a set amount of the starter culture and adding fresh water and fresh flour, though it can also refer to feeding a leftover portion of raw bread dough for the next bread-making (Urien et al., 2019). The re-feeding process may also be referred to as backslopping, propagating, or refreshing the starter. There are two ways to describe the re-feeding practices of sourdough starters: (1) the quantity of starter used during refreshments and, (2) the quantity of starter used in bread products. Differences among starter feeding practices around the world are not well-documented, and different amounts of starter may be used in different bread recipes depending on the final bread product to be made (e.g., a loaf versus a bagel) or the timeline of production. Bakers may also re-feed their starter with a smaller ratio of starter to fresh flour and water in order to compensate for a rapidly-fermenting, warm environment (seasonal adjustments). Nonetheless, the proportion of sourdough starter in bread can strongly influence a bread’s aromatic profile and using more starter in a bread recipe can yield more acidic bread doughs (Hansen & Hansen, 1996; Pétel, Onno & Prost, 2017). The proportion of starter used in a bread recipe has also been observed to positively correlate with yeast cell density and ethanol production (Lattanzi, Minervini & Gobbetti, 2014). Interestingly, Hansen & Hansen (1996) described how panelists liked bread with different amounts of starter, depending on what the starter was inoculated with, suggesting that starters with different ecological profiles can in fact have noticeably different technical and sensory properties.

### Environment

The exact location that a sourdough starter is made in or stored in may impact its microbial ecology. Many sourdough starter experiments have documented microbial and aromatic differences between sourdough starters made in laboratory versus bakery settings (Scheirlinck et al., 2007; Vrancken et al., 2010; Minervini et al., 2012b; Di Cagno et al., 2014; Harth, Van Kerrebroeck & De Vuyst, 2016). In addition, sourdough research to date has revealed broad trends in the presence and absence of species among starters maintained in the same country or geographic region (De Vuyst et al., 2014; Viiard et al., 2016; Liu et al., 2018; Fujimoto et al., 2019). Yet, we cannot explain patterns of species diversity in starters from different locations because we do not know how sourdough management methods vary per culture. We, like De Vuyst, Van Kerrebroeck & Leroy (2017), emphasize that in addition to sourdough ecology being influenced by *regional* geography, a starter’s ecology may be even more influenced by its *immediate* environment, such as local air quality and other products being stored near a starter. Comasio et al. (2020) exemplifies how a starter’s immediate environment can impact the starter’s ecology, as we discussed with the occurrence of AAB in starters stored in Lambic breweries (section 2.1). Examining the quantitative differences among starters from different locations has revealed a variety of ecologies and physiochemical profiles; but the qualitative methods by which starters are maintained around the world may be equally important for understanding sourdough.

### Other factors

In addition to the above factors, mixing practices, grain/flour milling practices, other storage practices, the starter’s immediate environment, the scale of bread production, and the use of adjuncts can also influence the microbial ecology of a sourdough starter (De Vuyst et al., 2014; Michel et al., 2019). Adjuncts, such as fruit juice, honey, and macerated fruit can influence the microorganisms in sourdough and result in a different metabolic potential of the starter (Minervini et al., 2016; Comasio, Van Kerrebroeck & De Vuyst, 2021). However, the effects of adjuncts on the sensory properties of Type I starters is minimally understood. Mixing practices influence the oxygen exposure and availability of a starter. Yeasts can use oxygen from kneading for their metabolism, and some yeasts, like *Pichia kudriavzevii,* even require oxygen (De Vuyst et al., 2014; De Vuyst et al., 2016). At the same time, oxygenation of the sourdough starter can also contribute to the oxidation of flour lipids and the conversion of ethanol to acetate by acetic acid bacteria, which can, in turn, impact the flavor of sourdough products (Pico, Bernal & Gómez, 2015; De Vuyst et al., 2016; De Vuyst, Van Kerrebroeck & Leroy, 2017). We found that many experiments that sampled bakery starters included little to no description of mixing practices (Scheirlinck et al., 2007; Osimani et al., 2009; Valmorri et al., 2010; Minervini et al., 2012a; Minervini et al., 2012b), while starters made in the laboratory setting were usually mixed continuously during fermentation (Van der Meulen et al., 2007; Weckx et al., 2010a; Weckx et al., 2010b; Ravyts & De Vuyst, 2011; Bessmeltseva et al., 2014; Harth, Van Kerrebroeck & De Vuyst, 2016; Van Kerrebroeck et al., 2018).

Other factors that may influence starter quality and ecology include water quality (Minervini et al., 2019), which differs per bakery depending on location and filtration systems. Additionally, feeding a starter with bare hands versus a mixer almost certainly introduces different microorganisms into the starter. Considering that females host a greater relative abundance of LAB on their hands (Fierer et al., 2008) including vaginal LAB, and that some vaginal LAB have competitive advantages over other sourdough species, there may even be a microbiological difference between starters that are routinely fed by men versus women (De Vuyst & Neysens, 2005; Grice & Segre, 2011; Michel et al., 2019; Reese et al., 2020). We suspect that if traditional, sourdough bread bakers are able to link their modifiable practices to observable food qualities, then they will be able to create more complex, unique bread products.

## Sourdough Sensory Quality

The land management and microbial ecology of sourdough starters influence various sensory qualities of sourdough starters and their subsequent bread products. Both are important to bakers: a starter’s sensory attributes guide management and baking practices, while bread attributes determine consumer demand and satisfaction. The sensory qualities that are most commonly described regarding sourdough starters and breads include their taste, aroma, texture, and visual characteristics (i.e., bubbles and height of a starter, crumb texture and structure of bread), which we showcased in Fig. 1; and all of these factors are key drivers in the liking and perception of food products (Lawless & Heymann, 2010a). Flavor encompasses taste as well as retronasal aromas, mouthfeel, and other sensory aspects of foods once they are in the mouth. A large body of research considers the volatile compounds of bread in general and sourdough bread in particular (Spicher & Nierle, 1988; Gänzle, Loponen & Gobbetti, 2008; Heenan et al., 2009; Pétel, Onno & Prost, 2017; Van Kerrebroeck et al., 2018; Landis et al., 2021). Flavor chemistry involves the quantification and identification of such volatile chemicals that give off unique tastes and aromas. While flavor chemistry research sheds light on the chemical basis of sourdough sensory quality, Heenan et al. (2009) suggest that bread flavors stem from the combinations of many different volatile organic compounds, not just a select few; and the human senses cannot distinguish the sensory characteristics of individual volatile organic compounds in mixtures of more than four compounds (Laing et al., 2002). In this way, sensory research is vital for investigating sourdough textures, flavors, and other sensory attributes in their naturally occurring form (Chambers & Koppel, 2013; Jeltema, Beckley & Vahalik, 2016). Yet, volatile information can still correlate with sensory evaluations (Heenan et al., 2009), making them useful in the initial development of a general lexicon—a set of descriptive terms—for describing sourdough aromas.

As is currently understood, sourdough volatiles and other flavor precursor compounds have been described to originate from three processes: (1) lipid oxidation, (2) browning reactions, and (3) fermentation (Pétel, Onno & Prost, 2017). Lipid oxidation is a volatile-generating pathway triggered by mixing and aeration of the sourdough culture, which introduces oxygen and aids in the oxidation of flour lipids. Browning reactions occur in ways that are very similar to fermentation reactions, wherein amines and saccharides react to form volatiles that are associated with roasty, caramel flavors and rich brown pigments on the crust of bread. Finally, diverse metabolic pathways associated with fermentation can produce many different compounds. Altogether, the flavor and sensory character of sourdough bread products is largely influenced by the microorganisms and land management practices that define a sourdough starter.

In considering the sensory research that has already been done on and proposed for bread (Katina et al., 2006; Heenan et al., 2009; Callejo, 2011; Pico, Bernal & Gómez, 2015), dried sourdough starters (Pétel et al., 2017), and *wheat-specific* sourdough (Lotong, Chambers IV & Chambers, 2000), there is room for our community to perform more sensory descriptive analyses (Lawless & Heymann, 2010b) of sourdough products. These may then be used to develop a modernized lexicon for both sourdough starters and their subsequent breads, similar to what was done by Kalanty (2015). With the increase in popularity of artisan sourdough baking, a modernized and broadly usable lexicon—analogous to the well-known Wine Wheel (Noble et al., 1984; Noble et al., 1987)—may help us to draw more tangible, observable connections between sourdough microbial ecology, land management, and sensory quality.

### Influence of microorganisms

As introduced, the influence of microorganisms on the flavor of sourdough starters and subsequent bread products is a growing area of fermentation research. Carbohydrates, amino acids, peptides, and lipids are the primary precursors of flavor in sourdough products, and both yeast and bacteria metabolize these compounds into volatile organic compounds that are responsible for unique tastes and aromas (Thiele, Gänzle & Vogel, 2002; Angelis & Gobbetti, 2004; Van der Meulen et al., 2007; De Vuyst et al., 2009; Gänzle, 2014). While the metabolic pathways for flavor formation by LAB and yeast are well-characterized (Hansen & Schieberle, 2005; Pétel, Onno & Prost, 2017), we don’t know which microbial species or strains are producing which specific metabolites at any given time. We do know generally that some specific yeasts are associated with specific volatiles and flavors. For example, *S. cerevisiae* has been linked to balsamic, malty, honey, rose, and buttery flavors, whereas *K. humilis* has been linked to fruity, green flavors (Liu et al., 2018). But, even knowing that *K. humilis* is acid or cold tolerant, we cannot say that all cold-fermented starters will smell “fruity”. Different species of yeast also produce different amounts of CO_2_ (Häggman & Salovaara, 2008b; Tsegaye et al., 2018), which can affect the texture and volume of sourdough bread products. Together, these examples indicate that yeasts make important contributions to the sensory character of Type I sourdough starters and breads.

The relative abundance of LAB in sourdough starters, compared to other bacterial taxa, has also been observed to influence flavor (Reese et al., 2020), and some specific bacterial species have been linked to sour, roasty, and generally “improved” flavors (Thiele, Gänzle & Vogel, 2002; De Vuyst & Neysens, 2005; Ravyts & De Vuyst, 2011). There is also evidence to suggest that bacteria such as *Leuconostoc mesenteroides* and other LAB species can produce dextrans and diacetyl, contributing enhanced bread texture and buttery flavors, respectively (Decock & Cappelle, 2005; Lacaze, Wick & Cappelle, 2007; Chen, Levy & Gänzle, 2016; Comasio et al., 2019; Comasio, Van Kerrebroeck & De Vuyst, 2021).

Most of the research that attempts to connect sourdough ecology to sensory quality describes the most common sourdough species through inoculated, culture-based approaches. These methods generally do not consider the impact of spontaneously occurring organisms, co-fermentation, or different traditional management practices on the sourdough system. Thus, we propose that new research should examine the microbial ecology and sensory quality of authentic sourdough products (i.e., bread and starter cultures) within the craft culinary industry. In particular, assessing sourdough samples from across the United States would supplement existing sourdough research, which focuses on bread and/or starters from European countries. We also suggest that starter and bread product sensory analyses could be conducted in more realistic settings, such as “bread festivals”, which bring together professional bakers and their products for a short amount of time and so provide samples and assessors in contextual conditions. Likewise, these methods would yield a large sample of starters and products, contrasting current research which is based mostly on convenience (i.e., samples of bakery-sampled starters and inoculated breads) (Scheirlinck et al., 2007; Minervini et al., 2012a; De Vuyst et al., 2014).

### Influence of land management

Given that land management practices directly influence microbial ecology, and that microbial ecology largely influences the flavors of fermented products, it is likely that maintenance practices closely relate to sensory quality differences in sourdough products. In considering the type of flour that is fed to a starter, there is evidence that the lipid fraction of grain and ash content in grain are related to lipid-based volatiles in a starter (Damiani et al., 1996; Gänzle, Vermeulen & Vogel, 2007; Ravyts & De Vuyst, 2011). Thus, non-traditional flours with unique lipid profiles may lead to unique tasting breads. Likewise, there are substantial differences in the volatiles that are associated with wheat versus rye sourdoughs (Hansen & Schieberle, 2005; Pétel, Onno & Prost, 2017). Beyond the sensory differences among grain species, there is evidence that even different varieties of wheat yield sourdough products with different sensory characteristics (Coda et al., 2010). A productive area of future research would be assessing the sensory quality of sourdough products made with non-traditional flour varieties and adjuncts, which have been previously described for their potential influence on microbial ecology. Compared to the literature on the microbial ecology of sourdough starters with non-traditional flour, there is much less knowledge regarding the sensory quality of such starters and their subsequent bread products.

Other land management practices worth discussing for their influence on the flavor chemistry and sensory quality of sourdough products include temperature, refeeding practices, and hydration. Higher temperatures are thought to favor lactic acid production and are associated with more flower and fruit aromas, while lower temperatures have been linked to acetic acid, as well as rancid flavors from butanoic acid (Siepmann et al., 2019). Comparatively, prolonged fermentation time has been connected to burnt, bitter, or unpleasant flavors and excessive organic acid content (Meignen et al., 2001; Thiele, Gänzle & Vogel, 2002; Van Kerrebroeck et al., 2018). Similarly, yeasts produce more ethanol under acid stress, suggesting that under-fed starters may display themselves by being tart, vinegary, or alcohol-like (Guerzoni et al., 2007). Given the positive correlation between fermentation time and organic acid production (Fig. 3B), and the potential for organic acid to inhibit yeast, fermentation time and feeding frequency may closely impact yeast-associated flavors (Gobbetti et al., 1995; Narendranath, Thomas & Ingledew, 2001; De Vuyst et al., 2014; Van Kerrebroeck et al., 2018). Similarly, the amount of starter that is used during re-feedings and in subsequent bread products can also affect the sensory quality of sourdough starters by contributing more or less fermenting cells (Zolfaghari et al., 2017). Using more starter during re-feedings or in bread-baking will allow for a quicker fermentation and can lead to more pungent flavors in the final bread product, which we described in section 3.5. In addition, there is some evidence to suggest that hydration affects the characterization of volatile organic compounds in sourdough starters (Gobbetti et al., 1995; Di Cagno et al., 2014). Future sensory research should seek to examine the organoleptic qualities of starters and subsequent breads that are made according to experimentally modified but realistic land management practices (i.e., the range of fermentation temperature, feeding protocol, and hydration used by bakers) in order to identify which practices make the largest impact on flavor, texture, color, and other quality outcomes.

## Calls for Future Work

### Rheology

Rheology describes the study of the “flow of matter”, or raw dough in the case of sourdough bread-baking. The hydration of a sourdough starter and any subsequent sourdough bread products will closely influence the flow of the sourdough bread system, which can ultimately affect the structural and textural quality of final bread products (Jahromi et al., 2014). The impact of microbial ecology on the rheology of sourdough and final bread quality is of emerging interest because there is evidence that sourdough fermentation improves the elasticity, viscosity, and extensibility of dough compared to dough leavened primarily with baker’s yeast (Clarke et al., 2004; Häggman & Salovaara, 2008b; Gänzle, Loponen & Gobbetti, 2008; Bender et al., 2018; Gaglio et al., 2018; Yildirim-Mavis et al., 2019). Like the sensory research described in section 4.1, most rheological research to date involves the use of *laboratory inoculated* starters. Thus, there is little to no research assessing the rheological characteristics of *spontaneous* sourdough starters maintained under different conditions. The rheology of bread dough closely relates to the structure of the gluten network; so, linking microbial ecology to sourdough bread rheology may help scientists better understand how microorganisms affect the gluten content of sourdough breads or how non-gluten flours can be optimally used in sourdough starters considering their sensory challenges (Moroni et al., 2012; Coda et al., 2014). Notably, there is evidence that *Limosilactobacillus reuteri* and *Lactiplantibacillus plantarum* produce exopolysaccharides, which can serve as hydrocolloids to improve the structure of gluten-free breads (Galle & Arendt, 2014). What role could microorganisms play in lowering the gluten content of sourdough breads? More research into the relationship between microorganisms, rheology, and gluten content may help us better understand and optimize the nutritional quality of sourdough bread (Roesler, 2019).

### Defining traditional sourdough methods

In sourdough research, we have seen two methods of studying sourdough starters to date: (1) sampling starters from various bakeries (Valmorri et al., 2010; Minervini et al., 2012b; Di Cagno et al., 2014), or (2) making starters in laboratory settings according to prior sourdough experimental methods (Van der Meulen et al., 2007; Coda et al., 2010; Vrancken et al., 2010; Bessmeltseva et al., 2014). Because no scientific literature has ever assessed culinary practices related to the production of sourdough starters by region, we hypothesize that sourdough production practices in the literature may not accurately represent “wild” sourdough in the craft, culinary industry. To date, ample research has described sourdough starter production in bakeries in ways that are not truly reproducible in practice, at an artisan scale, or for the production of baked products (Catzeddu et al., 2006; Osimani et al., 2009; Vrancken et al., 2010; Minervini et al., 2012a; Minervini et al., 2012b; Viiard et al., 2016; Harth, Van Kerrebroeck & De Vuyst, 2016; Urien et al., 2019; Comasio et al., 2020). Of the experimental papers that we reviewed, 66.2% (55/83) failed to note the amount of starter that they used when re-feeding the sourdough culture; though De Vuyst, Van Kerrebroeck & Leroy (2017) reports the percentage of inoculum for to be between 5–20% (m/v) and Minervini et al. (2014) reports the percentage of inoculum to be between 10–40% (m/v), both for Type 1 starters. Osimani et al. (2009) describe how “the length of the sourdough fermentation time and temperature varied considerably among the nine bakeries, according to the particular cycle of production”. While we are not suggesting that all artisanal, traditional sourdough bakers follow the same rigid practices, we do propose that new sourdough research efforts seek to investigate traditional, diverse sourdough practices in bakeries. Qualitative research methods would help to paint a thick, rich description (Creswell & Poth, 2018) of common baking practices related to sourdough, similar to recent work by Urien et al. (2019) and Michel et al. (2019). We suspect that there are likely many more undisclosed practices that contribute to the ecological differences of sourdough starters and the organoleptic quality of sourdough breads.

## Conclusions

The sourdough ecosystem is a complex culture of highly specialized microorganisms that largely determine the quality, structure, and function of sourdough cultures in subsequent bread products. While making and using a sourdough starter is largely subjective, there is a growing body of evidence to suggest that sourdough practices correlate to certain microbial, sensorial, and tactile outcomes. The succession of species during the initial propagation of a starter and during continued maintenance of a starter are unique to not only individual bakeries around the world, but also to the scientists working on them. In order to conclusively identify sourdough characteristics, their causal factors, and their effective outcomes in culinary applications, more research is needed. Such research should seek to better understand sourdough species relative to flour type and traditional bakery propagation practices with controlled and reproducible practices, in relevant settings. Likewise, future research should also seek to connect microbial ecology to the sensory and technical qualities of sourdough products. Even though the use of sourdough starters dates back to ancient times, the resurgence of baking with sourdough is opening up new culinary and agricultural ventures that encourage the use of local resources, but which also require a larger understanding of the science within.

## Additional Information and Declarations

### Data Availability

The following information was supplied regarding data availability: This is a Literature Review; there are no raw data or code.
